# Sleep-related problems among patients with rheumatoid arthritis in the World Health Organization Eastern Mediterranean region: a systematic review and meta-analysis

**DOI:** 10.3389/fpsyt.2026.1786989

**Published:** 2026-05-29

**Authors:** Mohammad Mustafa

**Affiliations:** Department of Medicine, University of Jeddah, Jeddah, Saudi Arabia

**Keywords:** Eastern Mediterranean region, insomnia, meta-analysis, obstructive sleep apnea, prevalence, rheumatoid arthritis, risk factors, sleep quality

## Abstract

**Background:**

Sleep-related problems are common among patients with rheumatoid arthritis (RA) and contribute substantially to disease burden and reduced quality of life. Evidence from the World Health Organization Eastern Mediterranean Region (WHO EMRO) remains fragmented, with variability in reported prevalence, sleep constructs assessed, and associated risk factors. This study aimed to systematically review and meta-analyze the prevalence and correlates of sleep-related problems among adults with RA in WHO EMRO countries.

**Methods:**

A systematic search of PubMed, Scopus, Web of Science, Embase, CINAHL, and Google Scholar was conducted to identify observational studies reporting sleep-related outcomes among adults (≥18 years) with RA in WHO EMRO countries from inception to 27 July 2025. Study screening, data extraction, and quality appraisal using Joanna Briggs Institute (JBI) checklists were conducted primarily by a single author, with methodological oversight and consultation from senior collaborators. Eligible studies reported prevalence or sufficient data to calculate prevalence of specific sleep-related constructs, including subjective sleep quality, insomnia symptoms, daytime sleepiness, or obstructive sleep apnea. Random-effects meta-analysis (DerSimonian-Laird) was used to pool prevalence estimates and odds ratios (ORs) for associated risk factors. Heterogeneity was assessed using the I² statistic and Cochran’s Q test. Subgroup analyses were conducted by sleep construct, assessment method, and country. Publication bias was evaluated using funnel plots, Egger’s test, and Begg’s test, with cautious interpretation due to the small number of studies.

**Results:**

Ten studies met inclusion criteria for qualitative synthesis, and six studies (n = 2,315 participants) were included in the quantitative meta-analysis. The pooled prevalence of sleep-related problems was 60.9% (95% CI: 56.5%–65.2%), with substantial heterogeneity (I² = 88%, p < 0.001), reflecting differences in sleep constructs and assessment methods. Subgroup analyses yielded pooled prevalence estimates of 58% for insomnia symptoms, 65% for obstructive sleep apnea-related measures, and 61% for poor subjective sleep quality (PSQI above cut-off), with no statistically significant differences between subgroups. Among associated factors, depression showed the strongest association with sleep-related problems (OR = 2.65; 95% CI: 1.87–3.75), followed by pain (OR = 2.18; 95% CI: 1.68–2.84), fatigue (OR = 1.91; 95% CI: 1.45–2.52), female gender (OR = 1.67; 95% CI: 1.25–2.23), and older age (OR = 1.42; 95% CI: 1.12–1.80). Heterogeneity for risk factor analyses ranged from low to moderate. Publication bias assessments did not indicate statistically significant small-study effects but were underpowered.

**Conclusion:**

Sleep-related problems—assessed using heterogeneous subjective and objective measures—are highly prevalent among patients with RA in WHO EMRO countries with available data. Given substantial clinical and methodological heterogeneity and the limited number of contributing countries, findings should be interpreted as reflecting overall sleep-related burden rather than the prevalence of a single clinical disorder. Routine screening, multidisciplinary management, and culturally adapted interventions are recommended. Future large-scale, longitudinal studies using standardized diagnostic criteria across a broader range of WHO EMRO countries are needed to strengthen the evidence base.

**Systematic review registration:**

https://www.crd.york.ac.uk/PROSPERO/view/CRD420251109906, identifier CRD420251109906.

## 1 Introduction

Rheumatoid arthritis (RA) is a chronic systemic autoimmune disease characterized by persistent synovial inflammation, progressive joint damage, and extra-articular manifestations, leading to impaired physical function and reduced quality of life (1, 2). Globally, RA affects approximately 0.5%–1% of the adult population and occurs more frequently among women and older adults (3). Increasing attention has been directed toward comorbid conditions in RA, particularly sleep-related problems, which contribute substantially to physical disability, psychological distress, and overall disease burden (4).

Sleep-related problems—including insomnia symptoms, poor subjective sleep quality, obstructive sleep apnea (OSA), restless legs syndrome (RLS), and periodic limb movement disorder—are frequently reported among individuals with RA, with prevalence estimates commonly ranging from 50% to 70% across different populations and assessment methods (5, 6). These sleep outcomes represent distinct clinical and subclinical constructs rather than a single unified disorder, and their reported prevalence varies according to diagnostic definitions and measurement tools.

The relationship between RA and sleep-related problems is complex and bidirectional. Systemic inflammation mediated by pro-inflammatory cytokines such as interleukin-6 (IL-6) and tumor necrosis factor-α (TNF-α) can disrupt sleep architecture and promote non-restorative sleep, while chronic sleep disturbance may in turn exacerbate inflammatory activity (7, 8). Pain interferes with sleep initiation and maintenance and contributes to central sensitization, reinforcing a cycle of worsening symptoms. Psychological comorbidities, particularly depression and fatigue, are common in RA and are both contributors to and consequences of impaired sleep, further diminishing functional status and quality of life (9). These interrelated mechanisms underscore the need for integrated management strategies that address pain, psychological distress, and sleep-related symptoms concurrently.

The World Health Organization Eastern Mediterranean Region (WHO EMRO) includes countries across the Middle East, North Africa, and parts of West and Central Asia and is characterized by considerable genetic, environmental, and sociocultural diversity. These contextual factors may influence both the epidemiology of RA and the occurrence, recognition, and reporting of sleep-related problems. Studies conducted in selected WHO EMRO countries—most notably Saudi Arabia, Egypt, Iran, and Jordan—have reported a high burden of sleep-related problems among patients with RA (10–12). However, the available literature is heterogeneous, often limited by small sample sizes, cross-sectional designs, and variability in sleep constructs assessed and measurement instruments used. Moreover, potential determinants such as disease activity, medication exposure (e.g., corticosteroids), psychological distress, and sociodemographic characteristics differ across populations within the region, and their associations with sleep outcomes have not been synthesized systematically.

Focusing on WHO EMRO is particularly relevant given the region’s distinct epidemiological and health-system characteristics, including high rates of consanguinity in some countries, unequal access to specialized rheumatology and sleep medicine services, and limited availability of advanced diagnostic tools such as polysomnography. In addition, ongoing socioeconomic and geopolitical challenges in parts of the region may contribute to heightened psychosocial stress and disruptions in healthcare delivery (13–15). Cultural attitudes toward sleep, pain expression, mental health, and healthcare-seeking behavior may further shape how sleep-related symptoms are experienced and reported (16). Despite these considerations, no prior systematic review or meta-analysis has comprehensively examined sleep-related problems and their associated factors among patients with RA across WHO EMRO countries.

In light of these gaps, the present study aims to systematically review and meta-analyze the available evidence to (1) estimate the pooled prevalence of sleep-related problems among adults with RA in WHO EMRO countries with published data, and (2) identify key demographic, clinical, and psychosocial factors associated with these outcomes. By synthesizing region-specific evidence, this review seeks to inform clinical practice and support the development of culturally appropriate screening and management strategies for sleep-related problems in RA within the WHO EMRO context.

## 2 Methods

### 2.1 Study design and protocol registration

This systematic review and meta-analysis was conducted in accordance with the Preferred Reporting Items for Systematic Reviews and Meta-Analyses (PRISMA) 2020 guidelines (**Supplementary File 1**) (17). The study protocol was registered prospectively with PROSPERO (CRD420251109906). The final literature search was completed on 27 July 2025.

### 2.2 Eligibility criteria

Studies were selected according to the following predefined criteria:

- Population: Adults (≥18 years) with rheumatoid arthritis (RA). RA diagnosis was a mandatory inclusion criterion; however, the specific diagnostic or classification criteria used (e.g., 2010 ACR/EULAR criteria or clinical diagnosis by a rheumatologist) varied across studies and were not consistently reported. Measures of disease activity (e.g., DAS28) were extracted where available but were not considered diagnostic criteria.
- Outcome: Sleep outcomes were defined according to the specific sleep-related construct assessed by each instrument, including:
  - subjective sleep quality [e.g., Pittsburgh Sleep Quality Index (PSQI)],
  - insomnia symptoms (e.g., Insomnia Severity Index, Athens Insomnia Scale),
  - daytime sleepiness (e.g., Epworth Sleepiness Scale),
  - restless legs syndrome (validated RLS scales), and
  - obstructive sleep apnea (screening questionnaires or objective polysomnography).

For synthesis purposes, the term sleep-related problems was used as an umbrella descriptor encompassing these distinct constructs. Given the inherent clinical and methodological heterogeneity, separate subgroup analyses were prespecified by sleep construct and assessment modality. When multiple sleep outcomes were reported within the same study population, only one prevalence estimate per study—representing the most inclusive sleep construct—was included in the primary pooled analysis to avoid double counting. Disorder-specific prevalence estimates were synthesized only within their respective subgroup analyses.

- Study design: Observational studies (cross-sectional, case-control, or cohort).
- Setting: Studies conducted in countries classified under the World Health Organization Eastern Mediterranean Region (WHO EMRO). Eligible countries included Afghanistan, Bahrain, Djibouti, Egypt, Iran, Iraq, Jordan, Kuwait, Lebanon, Libya, Morocco, Oman, Pakistan, Palestine, Qatar, Saudi Arabia, Somalia, Sudan, Syria, Tunisia, United Arab Emirates, and Yemen. This WHO EMRO country list was applied consistently in the eligibility criteria and search strategies.
- Language: Articles published in English or Arabic.
- Exclusions: Case reports, reviews, editorials, randomized controlled trials, conference abstracts without full data, and grey literature. Conference abstracts and grey literature were excluded because they generally lack sufficient methodological detail and complete outcome data required for reliable quality appraisal and quantitative synthesis.

### 2.3 Information sources and search strategy

A comprehensive literature search was conducted in PubMed/MEDLINE, Scopus, Web of Science, Embase, CINAHL, and Google Scholar from inception to 27 July 2025. The search strategy was explicitly aligned with the WHO EMRO definition and incorporated the full list of EMRO country names to ensure exhaustive retrieval. Database-specific search strings, exactly as executed, are provided in **Supplementary File 2** to ensure transparency and reproducibility.

### 2.4 Study selection and data extraction

All retrieved records were imported into EndNote for de-duplication. Title/abstract screening, full-text assessment, and data extraction were conducted primarily by a single author. Although formal dual independent screening was not performed, methodological oversight and consultation with senior collaborators were undertaken for eligibility decisions and data verification when needed. This approach is acknowledged as a limitation.

Data were extracted using a pre-piloted standardized form, capturing study design, country, sample size, age and sex distribution, RA diagnostic approach, sleep-related construct and assessment tool, prevalence estimates, and effect measures for associated risk factors (adjusted or unadjusted odds ratios). Sleep outcomes were recorded using standardized terminology reflecting the exact construct measured, rather than nonspecific umbrella labels.

### 2.5 Quality assessment

Methodological quality was assessed using the Joanna Briggs Institute (JBI) critical appraisal checklists appropriate to each study design (18). Each checklist item was scored as “Yes,” “No,” or “Unclear” in accordance with JBI guidance. Overall quality scores were calculated by summing items rated “Yes” and classified as high quality (8–9 items), moderate quality (5–7 items), or low quality (≤4 items). No additional or hybrid quality categories were applied.

### 2.6 Certainty of evidence (GRADE)

The certainty of evidence for the primary outcome (overall prevalence of sleep-related problems) and key associated factors (pain, depression, fatigue, age, and sex) was assessed using the GRADE approach. Evidence was initially rated as low due to the observational design and was further downgraded as appropriate for risk of bias, inconsistency, indirectness, imprecision, and publication bias. A Summary of Findings table was generated (**Table 1**).

**Table 1:** Characteristics of included studies.

Author(s)	Year	Country	Study design	Sample size (RA patients)	Mean age (years)	% Female	Sleep-related construct assessed	Sleep assessment tool(s)	Prevalence/Key findings	Risk factors analyzed
Brahem M et al. (26)	2024	Tunisia	Cross-sectional	100	53.2 ± 11.2	89%	Daytime sleepiness; subjective sleep quality	ESS, PSQI	28% had daytime sleepiness (ESS ≥ 11); 51% had poor sleep quality (PSQI > 5.5)	Disease activity (DAS28), pain (VAS), disability (HAQ), joint counts, corticosteroid use
Abdelrahman MSI et al. (12)	2024	Egypt	Case-control	30 RA patients; 30 healthy controls	50 ± 9.37	90%	Insomnia symptoms; subjective sleep quality; daytime sleepiness	ISI, ESS, PSQI	RA patients had significantly worse insomnia and sleep quality than controls	Disease activity, anxiety, depression, quality of life
Alanazi HQ et al. (22)	2024	Saudi Arabia	Multicenter cross-sectional	1,584	34 (mean)	84.8%	Subjective sleep disturbance	PSQI	63.6% reported poor sleep quality	Depression, anxiety, HRQoL
Gouda W et al. (10)	2023	Egypt	Multicenter cross-sectional	247	Not reported	77%	Subjective sleep quality; OSA risk	PSQI, ESS, Berlin Questionnaire	Poor sleep quality associated with DAS28-CRP, HAQ-DI, fatigue; OSA risk predicted by disability and fatigue	Disease activity, disability, fatigue, depression
Wali SO et al. (19)	2022	Saudi Arabia	Cross-sectional	60	49.9 ± 13.5	93.3%	Obstructive sleep apnea	Berlin Questionnaire, PSG	69% confirmed OSA among participants undergoing PSG	BMI, airway anatomy
Hammam N et al. (24)	2020	Egypt	Cross-sectional	115	Not reported	Not reported	Subjective sleep disturbance; fatigue	PSQI, BDI, MAF	RA patients had worse sleep and fatigue than controls; sleep disturbance correlated with fatigue	Depression, disease activity
Wali SO et al. (20)	2020	Saudi Arabia	Prospective (PSG-based)	199 (110 underwent PSG)	48.9 ± 12.7	94%	Obstructive sleep apnea	Berlin Questionnaire, ESS, PSG	58.1% had OSA (AHI ≥ 5); 22.9% had moderate-severe OSA	BMI, disease activity (DAS28)
Mustafa M et al. (21)	2019	Saudi Arabia	Clinic-based survey	101	48.7 ± 14.6	95%	Insomnia symptoms; daytime sleepiness; OSA risk; RLS	Berlin Questionnaire, ESS, AIS, RLS scale	Insomnia 63%; RLS 63%; OSA risk 37%; EDS 20%	Disease activity (DAS28), HAQ
Alsharaki A et al. (23)	2018	Egypt	Case-control	30 RA patients; 30 controls	Not reported	Not reported	Obstructive sleep apnea	ESS, overnight PSG	60% had OSA; mean AHI 23.4 ± 26.3/h	BMI, neck circumference, ESR, CRP
Purabdollah M et al. (25)	2015	Iran	Descriptive-correlational	210	48.4 ± 12.9	~74%	Daytime sleepiness; subjective sleep quality	ESS, SDQ, SF-36	Mean ESS = 13.1; poor sleep associated with reduced QoL	Pain, sleep disturbance, QoL

### 2.7 Data synthesis and statistical analysis

Meta-analyses were performed using Comprehensive Meta-Analysis (version 4). Prevalence estimates were pooled using a random-effects model (DerSimonian-Laird) with Freeman-Tukey double arcsine transformation and back-transformation for presentation. Odds ratios (ORs) for associated risk factors were pooled using the generic inverse-variance method under a random-effects framework.

Given the heterogeneity of sleep constructs and assessment methods, analyses were stratified by sleep construct and assessment modality (patient-reported measures versus objective or physician-diagnosed outcomes). Heterogeneity was assessed using the I² statistic, Cochran’s Q test (*p* < 0.10), and between-study variance (τ²). Prediction intervals were calculated but interpreted cautiously due to the limited number of studies.

Subgroup analyses were prespecified by sleep construct, assessment method, country (where ≥2 studies), and study quality. Sensitivity analyses excluded low-quality studies. Publication bias was evaluated using funnel plots, Egger’s regression test, and Begg’s test, with cautious interpretation due to limited statistical power.

## 3 Results

### 3.1 Study selection

**Figure 1:**
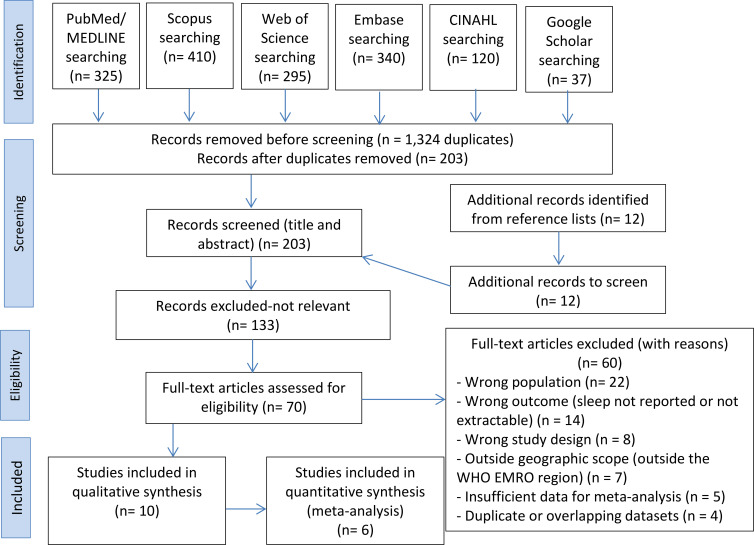
PRISMA 2020 flow diagram.

Database searches identified 1,527 records, and an additional 12 records were identified through manual reference list screening. After de-duplication, 203 unique records identified from databases remained and were screened by title and abstract. The 12 records identified through manual searching were screened separately and subsequently assessed for eligibility, as depicted in the PRISMA 2020 flow diagram (**Figure 1**).

Following screening, 70 full-text articles (58 from databases and 12 from manual searching) were assessed for eligibility. Of these, 60 studies were excluded for predefined reasons, including ineligible populations, outcomes not reporting sleep-related measures or prevalence data, studies conducted outside the WHO EMRO region, non-observational designs, conference abstracts without full data, and duplicate or overlapping datasets.

Ultimately, 10 studies met the inclusion criteria for qualitative synthesis, of which 6 provided sufficient data for inclusion in the quantitative meta-analysis. The remaining 4 studies were included in the systematic review but excluded from the meta-analysis due to insufficient or non-comparable quantitative data. All study counts and screening stages reported in the text are consistent with the PRISMA 2020 flow diagram (**Figure 1**).

### 3.2 Study characteristics

The included studies were conducted in WHO EMRO countries with available eligible data, primarily Saudi Arabia and Egypt, with additional studies from Iran and Tunisia. No eligible studies were identified from several other WHO EMRO countries, reflecting limited geographic coverage within the region.

Sleep outcomes were classified according to the specific sleep-related construct assessed, including subjective sleep quality, insomnia symptoms, daytime sleepiness, restless legs syndrome, and obstructive sleep apnea (OSA). Studies that originally used non-specific terminology (e.g., “general sleep disorders”) were reclassified based on the validated instruments employed (e.g., PSQI for subjective sleep quality, ESS for daytime sleepiness).

Sample sizes varied substantially, ranging from 30 participants in small case-control studies (23) to 1,584 participants in large multicenter surveys (22). Most studies employed cross-sectional designs, while one study adopted a prospective design incorporating polysomnography (PSG) for diagnostic confirmation of OSA (20). One included case-control study enrolled 30 patients with rheumatoid arthritis and 30 age- and sex-matched healthy controls (12), as reported in the original publication.

Rheumatoid arthritis was diagnosed using 2010 ACR/EULAR classification criteria in some studies (19, 24), or by clinical diagnosis by a rheumatologist in others (10, 21, 23, 25). Measures such as DAS28 were used to assess disease activity, not for diagnostic purposes (12, 21, 26).

Sleep-related problems were assessed using a range of validated instruments. OSA was evaluated using the Berlin Questionnaire, Epworth Sleepiness Scale (ESS), and overnight PSG where available (20, 23). Subjective sleep quality and insomnia symptoms were assessed using the Pittsburgh Sleep Quality Index (PSQI), Athens Insomnia Scale, and Insomnia Severity Index, with additional instruments measuring fatigue, depression, and health-related quality of life (10, 12, 22, 25).

Overall, the included studies demonstrate substantial methodological heterogeneity in both RA assessment and sleep-related outcome measurement, underscoring the need for standardized diagnostic criteria and harmonized sleep assessment approaches in future research (**Table 1**).

### 3.3 Methodological quality of included studies

Using the Joanna Briggs Institute (JBI) critical appraisal checklists, four studies were rated as high quality, four as moderate quality, and two as low quality, based strictly on the prespecified scoring thresholds.

Common methodological limitations included convenience sampling, limited reporting of non-responders, lack of multivariable adjustment for potential confounders, and, in some studies, incomplete reporting of rheumatoid arthritis diagnostic approaches.

Most studies employed appropriate sampling frames and clearly defined target populations, particularly the larger and multicenter investigations (10, 20, 22). In contrast, some smaller studies—such as those by Alsharaki et al. (23) and Purabdollah et al. (25)—relied on convenience sampling or lacked clearly defined recruitment strategies, which may limit external validity and generalizability (23, 25).

Sample size adequacy varied across studies. While several investigations recruited large or moderately sized cohorts, a few studies included relatively small samples, increasing the risk of limited statistical power and type II error (23). Measurement of sleep-related outcomes and RA was generally appropriate, with most studies using validated instruments such as the Pittsburgh Sleep Quality Index (PSQI), Epworth Sleepiness Scale (ESS), Berlin Questionnaire, and polysomnography (PSG) where feasible.

Potential confounding factors—including disease activity, fatigue, depression, and body mass index—were identified in most studies; however, only a subset applied multivariable analyses or other strategies to address confounding, which may affect internal validity (10, 12, 20).

Overall, methodological quality across the included studies was predominantly moderate, with a smaller number of high-quality studies and two studies rated as low quality due to limited reporting or insufficient control of bias and confounding. These findings highlight the need for more robust study designs, standardized reporting, and consistent analytical adjustment in future research on sleep-related problems among patients with rheumatoid arthritis (**Table 2**).

**Table 2:** Methodological quality of included studies based on JBI checklist.

Study (author, year)	Appropriate sampling frame	Appropriate sample size	Valid measurement of condition	Standardized assessment tool	Confounding factors identified	Strategies to deal with confounders	Appropriate statistical analysis	Overall quality rating
Brahem M et al., 2024 (26)	Yes	Yes	Yes	Yes	Yes	Unclear	Yes	Moderate
Abdelrahman MSI et al., 2024 (12)	Yes	Unclear	Yes	Yes	Yes	Yes	Yes	High
Alanazi HQ et al., 2024 (22)	Yes	Yes	Yes	Yes	Yes	Yes	Yes	High
Gouda W et al., 2023 (10)	Yes	Yes	Yes	Yes	Yes	Yes	Yes	High
Wali SO et al., 2022 (19)	Yes	Unclear	Yes	Yes	Unclear	No	Yes	Moderate
Hammam N et al., 2020 (24)	Yes	Unclear	Yes	Yes	Yes	No	Yes	Moderate
Wali SO et al., 2020 (20)	Yes	Yes	Yes	Yes	Yes	Yes	Yes	High
Mustafa M et al., 2019 (21)	Yes	Unclear	Yes	Yes	Yes	No	Yes	Moderate
Alsharaki A et al., 2018 (23)	No	Unclear	Yes	Yes	Unclear	No	Yes	Low
Purabdollah M et al., 2015 (25)	No	Yes	Unclear	Unclear	Yes	No	Yes	Low

Overall study quality was determined by summing Joanna Briggs Institute (JBI) checklist items rated as “Yes” (High: 8–9; Moderate: 5–7; Low: ≤4), in accordance with the prespecified criteria described in the Methods.

### 3.4 Pooled prevalence of sleep-related problems in RA patients

Six studies were included in the quantitative meta-analysis of prevalence. The pooled prevalence of sleep-related problems among patients with rheumatoid arthritis was 60.9% (95% CI: 56.5%–65.2%).

Substantial heterogeneity was observed across studies (I² = 88%; Q = 41.2, df = 5, p < 0.001), indicating considerable between-study variability beyond chance. This heterogeneity likely reflects clinical and methodological differences, including variation in the sleep-related constructs assessed (e.g., insomnia symptoms, obstructive sleep apnea, subjective sleep quality), assessment modalities (patient-reported outcome measures versus objective diagnostic tools such as polysomnography), and study populations and healthcare settings.

Because sleep-related problems in RA encompass distinct clinical and subclinical entities with differing pathophysiological mechanisms and diagnostic thresholds, the pooled estimate should be interpreted as an indicator of overall sleep-related burden, rather than the prevalence of a single, unified clinical disorder. Accordingly, disorder-specific and assessment-specific subgroup analyses provide more clinically meaningful interpretation of the findings.

**Figure 2:**
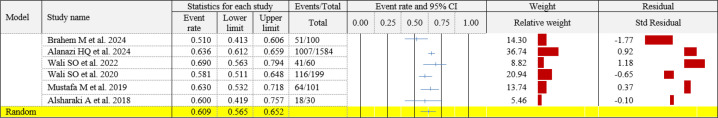
Pooled prevalence of sleep-related problems among patients with rheumatoid arthritis in WHO EMRO countries.

The prediction interval ranged from 49.2% to 71.6%, suggesting that the true prevalence of sleep-related problems in comparable RA populations may vary substantially across settings (**Figure 2**).

To avoid double counting, each study contributed a single, non-overlapping prevalence estimate to the primary analysis. When multiple sleep outcomes were reported, the estimate representing the broadest sleep-related construct was selected. Prevalence estimates derived from instruments such as the PSQI were interpreted as reflecting subjective sleep quality or perceived sleep disturbance, whereas estimates based on objective assessments or physician diagnosis reflected specific sleep disorders.

### 3.5 Heterogeneity and publication bias assessment

Substantial heterogeneity was observed among the included studies, as indicated by an I² value of 88% and a statistically significant Cochran’s Q test (Q = 41.2, df = 5, p < 0.001). The estimated between-study variance was moderate to high (τ² = 0.044; τ = 0.21), confirming the presence of considerable variability beyond chance.

Given this level of heterogeneity, a random-effects model (DerSimonian-Laird) was used for all primary analyses. The observed variability is likely attributable to clinical and methodological differences across studies, including heterogeneity in sleep-related constructs assessed (e.g., insomnia symptoms, obstructive sleep apnea, subjective sleep quality), diversity in assessment modalities (patient-reported outcome measures such as PSQI, ESS, and ISI versus objective measures such as polysomnography), and differences in study populations and healthcare contexts across WHO EMRO countries.

The prediction interval (49.2%–71.6%) further illustrates the potential dispersion of prevalence estimates in comparable future studies and reinforces the need for cautious interpretation of the pooled prevalence as an indicator of overall sleep-related burden rather than a single clinical outcome.

Publication bias was assessed using visual inspection of funnel plots, Egger’s regression test, and Begg’s rank correlation test. Neither Egger’s test nor Begg’s test indicated statistically significant small-study effects (p > 0.05 for both). However, given the small number of studies included in the meta-analysis (n = 6), these assessments are underpowered and should be interpreted with caution (**Table 3**).

**Table 3:** Heterogeneity and publication bias assessment for the pooled prevalence of sleep-related problems in patients with rheumatoid arthritis (WHO EMRO countries).

Random-effects meta-analysis														
Model	Effect size and 95% interval						Between-study		Other heterogeneity statistics					
Model	Number of studies (k)	Pooled prevalence		Lower limit		Upper limit	Upper limit	Tau (τ)	Tau-squared (τ²)	Cochran’s Q	Df (Q)	*p*-value (Q test)		I-squared (%)
Random	6	0.609		0.565		0.652	0.716	0.21	0.044	41.2	5	<0.001		88%
Publication Bias Assessment								Small-Study Effects and Robustness						
Test			Statistic		*p*-value		Interpretation	Test		Result			Interpretation	
Egger’s regression intercept			1.12 (SE = 1.05)		0.34		No significant asymmetry	Fail-safe N (classic)		~110 studies			Large number needed to nullify effect	
95% CI (Egger)			-3.86 to 1.62		*—*		Includes zero	Orwin’s Fail-safe N		~95 studies			Robust against null studies	
Begg’s test (Kendall’s tau)			-0.13		*0.71*		No significant bias	Trim-and-fill		0 studies imputed			No major publication bias detected	

Publication bias assessments were underpowered due to the small number of included studies (n = 6) and should therefore be interpreted with caution. The pooled prevalence represents overall sleep-related burden derived from heterogeneous sleep constructs rather than the prevalence of a single clinical sleep disorder.

**Figure 3:**
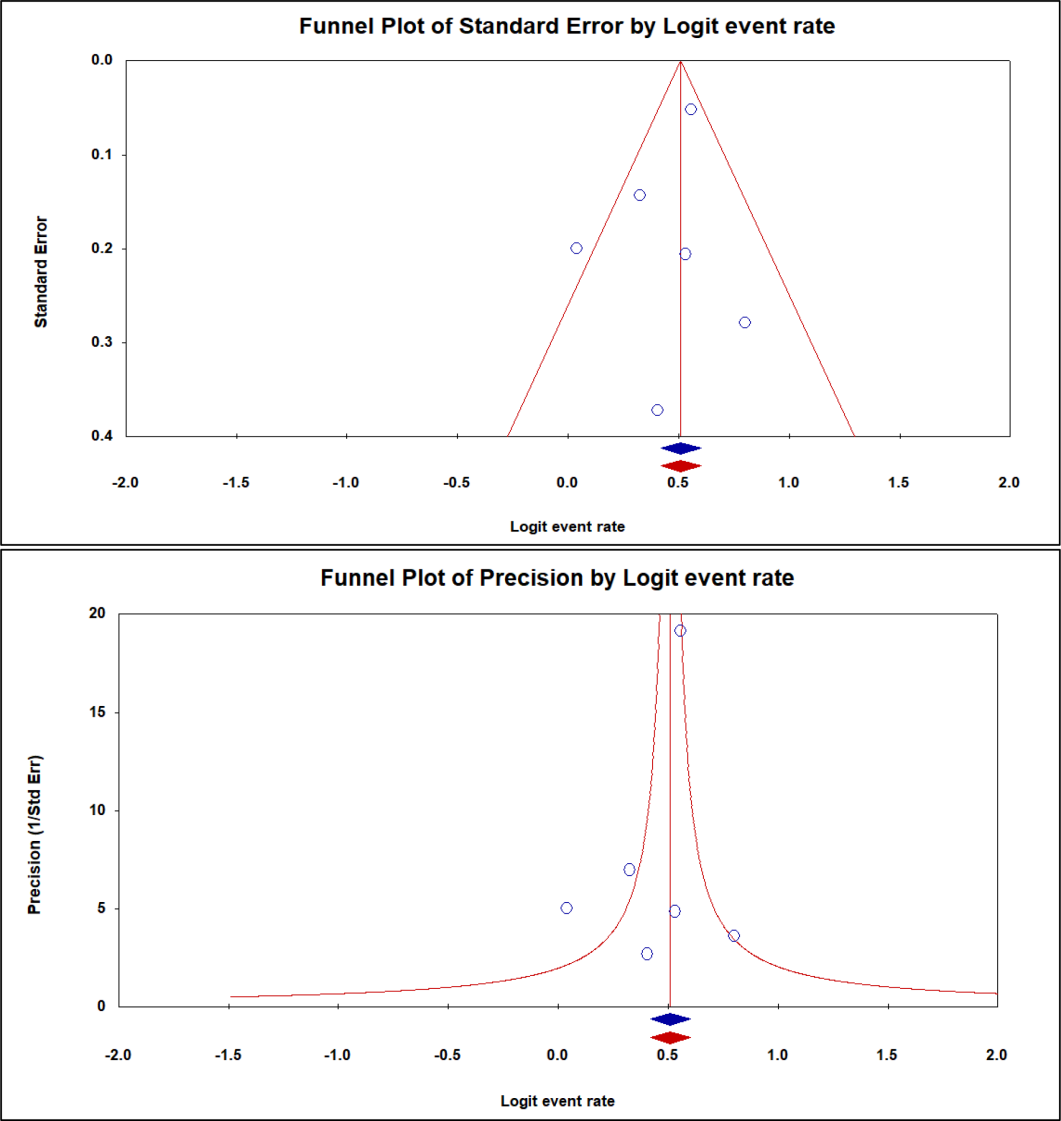
Funnel plot illustrating study precision and small-study effects among studies reporting the prevalence of sleep-related problems in patients with rheumatoid arthritis in WHO EMRO countries.

Visual inspection of the funnel plot suggested mild asymmetry; however, given the substantial between-study heterogeneity and the small number of included studies (n = 6), this pattern is more likely attributable to heterogeneity or small-study effects rather than true publication bias. Accordingly, results of funnel plot-based assessments should be interpreted with caution due to limited statistical power (**Figure 3**).

### 3.6 Subgroup analyses

To explore potential sources of clinical and methodological heterogeneity arising from differences in sleep-related constructs and assessment instruments, predefined subgroup analyses were conducted according to sleep construct, assessment modality, and country. Formal tests for subgroup differences were performed using the Q-test for between-group heterogeneity. Given that only six studies were included in the meta-analysis—and fewer contributed to individual subgroup comparisons—results should be interpreted cautiously due to limited statistical power and potential instability of between-study variance estimates.

Subgroup analyses were conducted independently using the relevant outcome from each study; participants were not double-counted across subgroups, as disorder-specific analyses were restricted to studies reporting the corresponding sleep construct.

When stratified by sleep construct, studies assessing insomnia symptoms (k = 3) yielded a pooled prevalence of 58% (95% CI: 54–62%), studies assessing obstructive sleep apnea (OSA) (k = 2) yielded a pooled prevalence of 65% (95% CI: 60–70%), and studies assessing subjective sleep quality using the PSQI above cut-off (k = 4) yielded a pooled prevalence of 61% (95% CI: 56–66%). Although point estimates differed, the overall test for subgroup differences was not statistically significant (p = 0.058). These findings underscore the clinical heterogeneity of sleep-related problems in RA and support disorder-specific interpretation rather than reliance on a single pooled estimate.

Stratification by assessment modality showed that studies using patient-reported outcome measures (PROMs), including PSQI, ESS, and ISI (k = 5), reported a pooled prevalence of 63% (95% CI: 59–67%), whereas studies using objective or physiologically based assessments (polysomnography or actigraphy) (k = 2) reported a pooled prevalence of 65% (95% CI: 60–70%). The between-group difference reached statistical significance (p = 0.031); however, this result should be interpreted cautiously due to the small number of studies employing objective measures. These findings suggest that assessment modality may influence prevalence estimates, with PROMs capturing subjective sleep disturbance and symptom burden, and objective tools identifying specific physiologically defined sleep disorders, such as OSA.

Country-level subgroup analysis demonstrated variability in prevalence estimates across WHO EMRO countries with available data. Studies from Saudi Arabia (k = 3) reported a pooled prevalence of 65% (95% CI: 60–70%), those from Iran (k = 2) reported 60% (95% CI: 55–65%), and those from Egypt (k = 2) reported 52% (95% CI: 47–57%). The overall test for subgroup differences was not statistically significant (p = 0.058), and these comparisons should be interpreted cautiously given the limited number of studies per country and potential residual confounding.

Other potentially relevant factors, including socioeconomic status, educational level, and detailed measures of RA disease activity, were inconsistently reported and could not be quantitatively synthesized. Nevertheless, where available, higher disease activity (DAS28/DAS28-CRP), greater disability (HAQ), and increased fatigue were consistently associated with worse sleep-related outcomes. These associations are examined further in the risk factor meta-analysis (**Table 4**).

**Table 4:** Subgroup analyses of sleep-related problem prevalence among patients with rheumatoid arthritis (WHO EMRO countries).

Subgroup category	Subgroup	k (studies)	Pooled prevalence (%)	95% CI (%)	Q-between p-value
Sleep Disorder Type	Insomnia	3	58	54–62	
	Obstructive Sleep Apnea (OSA)	2	65	60–70	
	Subjective sleep quality (PSQI > cut-off)	4	61	56–66	
Overall subgroup difference					0.058
Assessment Method	PROMs (PSQI, ESS, ISI)	5	63	59–67	
	Objective (PSG/actigraphy)	2	65	60–70	
Overall subgroup difference					0.031
Country	Saudi Arabia	3	65	60–70	
	Iran	2	60	55–65	
	Egypt	2	52	47–57	
Overall subgroup difference					0.058

Subgroup differences were assessed using between-group heterogeneity (Q-between) tests. Given the small number of studies within subgroups, p-values and pooled estimates should be interpreted cautiously. Prevalence estimates represent heterogeneous sleep-related constructs rather than a single clinically defined sleep disorder.

### 3.7 Risk factors associated with sleep-related problems

Meta-analysis of associated factors was restricted to variables reported in at least two studies using comparable effect measures. For each pooled estimate, the number of contributing studies (k) and heterogeneity statistics were calculated. Only studies reporting odds ratios (ORs) with 95% confidence intervals, or sufficient data for their calculation, were included; studies reporting non-comparable metrics (e.g., correlation coefficients or mean differences) were excluded from quantitative synthesis.

Among clinical factors, depression demonstrated the strongest association with sleep-related problems (k = 3; OR = 2.65; 95% CI: 1.87–3.75), with low heterogeneity (I² = 38%; Q = 3.21, p = 0.20). Pain was also strongly associated (k = 3; OR = 2.18; 95% CI: 1.68–2.84), with moderate heterogeneity (I² = 52%; Q = 4.17, p = 0.12). Fatigue showed a significant association as well (k = 2; OR = 1.91; 95% CI: 1.45–2.52), with low heterogeneity (I² = 25%; Q = 1.33, p = 0.25).

With respect to demographic factors, female sex was associated with increased odds of sleep-related problems (k = 3; OR = 1.67; 95% CI: 1.25–2.23), with low heterogeneity (I² = 29%; Q = 2.81, p = 0.25). Older age showed a modest but statistically significant association (k = 2; OR = 1.42; 95% CI: 1.12–1.80), with negligible heterogeneity (I² = 0%; Q = 0.84, p = 0.36).

**Figure 4:**
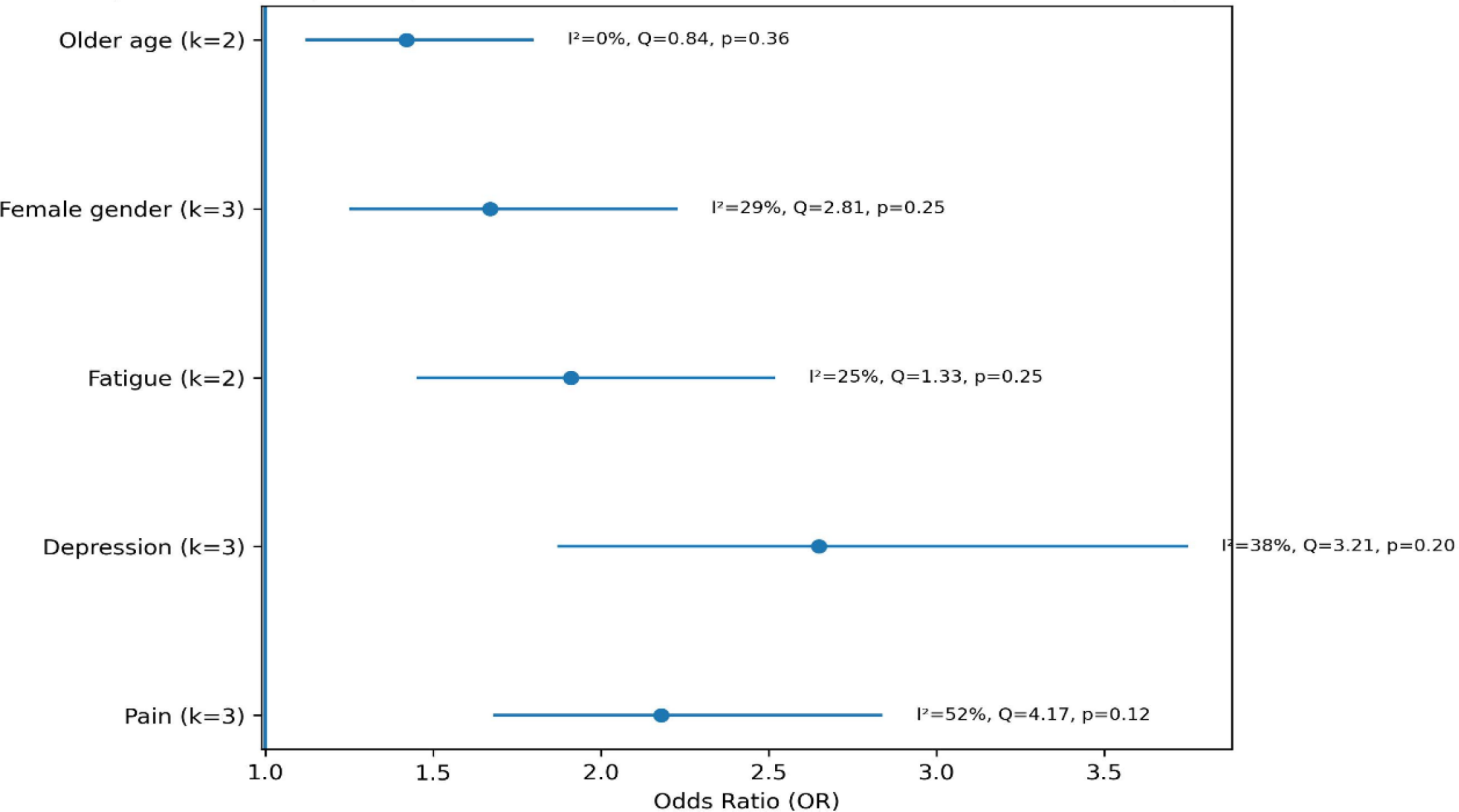
Forest plots of pooled odds ratios for factors associated with sleep-related problems among patients with rheumatoid arthritis in WHO EMRO countries.

Overall, heterogeneity across risk factor analyses ranged from low to moderate, and findings should be interpreted cautiously given the small number of contributing studies for several associations. Forest plots summarizing pooled estimates for each risk factor are presented in **Figure 4**.

### 3.8 Certainty of evidence (GRADE)

Using the GRADE framework, the overall certainty of evidence for the pooled prevalence of sleep-related problems among patients with rheumatoid arthritis was rated as low. This rating reflects the observational nature of the included studies and was further downgraded due to substantial heterogeneity across prevalence estimates and indirectness resulting from the pooling of clinically and methodologically heterogeneous sleep-related constructs and assessment modalities.

For associations between clinical and demographic factors and sleep-related problems, the certainty of evidence ranged from low to very low. Downgrading was primarily driven by risk of bias inherent to observational (predominantly cross-sectional) study designs, inconsistency across studies, and imprecision due to the small number of contributing studies for several pooled analyses. Additional downgrading was applied for potential residual confounding, as adjusted effect estimates were not consistently reported.

Although statistically significant associations were observed for depression, pain, fatigue, female sex, and older age, the limited certainty of evidence indicates that these findings should be interpreted cautiously and considered suggestive rather than confirmatory (**Table 5**).

**Table 5:** Certainty of evidence (GRADE) for sleep-related problems and associated factors in patients with rheumatoid arthritis (WHO EMRO countries).

Outcome	No. of studies (k)	Study design	Effect estimate (95% CI)	Certainty of evidence (GRADE)	Main reasons for downgrading
Prevalence of sleep-related problems (any construct)	6	Observational (cross-sectional)	60.9% (56.5%–65.2%)	Low	Serious inconsistency (I² = 88%); indirectness due to pooling heterogeneous sleep constructs and assessment methods
Depression (association with sleep-related problems)	3	Observational	OR = 2.65 (1.87–3.75)	Low	Risk of bias; imprecision (small k); potential residual confounding
Pain	3	Observational	OR = 2.18 (1.68–2.84)	Very low	Risk of bias; inconsistency (I² = 52%); residual confounding
Fatigue	2	Observational	OR = 1.91 (1.45–2.52)	Very low	Very serious imprecision (k = 2); risk of bias; limited generalizability
Female sex	3	Observational	OR = 1.67 (1.25–2.23)	Low	Risk of bias; residual confounding; limited number of studies
Older age	2	Observational	OR = 1.42 (1.12–1.80)	Very low	Serious imprecision (k = 2); risk of bias

Certainty ratings were assessed using the GRADE framework. All outcomes were initially rated as low due to observational study design and were downgraded further based on inconsistency, indirectness, imprecision, and potential residual confounding. Sleep-related problems encompass heterogeneous subjective and objective sleep constructs rather than a single clinically defined sleep disorder.

## 4 Discussion

This systematic review and meta-analysis synthesizes available evidence on sleep-related problems among patients with rheumatoid arthritis (RA) from WHO Eastern Mediterranean Region (EMRO) countries with published eligible studies, primarily Saudi Arabia and Egypt, with additional data from Iran and Tunisia. The findings indicate that sleep-related problems—encompassing heterogeneous constructs such as subjective poor sleep quality, insomnia symptoms, obstructive sleep apnea (OSA)-related measures, and daytime sleepiness—were reported in approximately 60.9% of patients with RA in the included studies. Given the clinical and methodological diversity of these outcomes, this pooled estimate should be interpreted as an indicator of overall sleep-related burden, rather than the prevalence of a single, clinically defined sleep disorder.

This overall burden is broadly consistent with reports from RA populations in other regions, underscoring impaired sleep as a common and clinically important comorbidity in RA (4, 27). Importantly, the observed burden likely reflects the combined influence of biological disease mechanisms, psychological comorbidities, and contextual health-system factors, rather than a uniform sleep pathology.

Beyond prevalence, this review identified several factors consistently associated with sleep-related problems. Depression demonstrated the strongest association, followed by pain and fatigue, highlighting the multifactorial nature of sleep impairment in RA. The strong relationship between depression and sleep problems is consistent with the well-established bidirectional interactions between mood disorders, systemic inflammation, and sleep regulation in RA (28, 29). Pain likely contributes directly to sleep disruption through nocturnal discomfort and fragmented sleep, while fatigue may function as both a consequence and a perpetuating factor of poor sleep quality. Demographic associations—specifically higher odds among women and older individuals—may reflect hormonal influences, cumulative disease burden, age-related changes in sleep architecture, and psychosocial vulnerability (30).

The substantial heterogeneity observed across studies was expected, given differences in sleep constructs assessed, measurement approaches, and study populations. Prevalence estimates derived from patient-reported outcome measures (PROMs), such as the PSQI, reflect subjective sleep disturbance and perceived sleep quality, whereas studies using objective assessments or physician diagnoses identify specific clinical disorders, such as OSA. These conceptual and diagnostic differences mean that a single pooled estimate inevitably masks disorder-specific variation. To address this, the primary analysis included only one prevalence estimate per study to avoid population overlap, while disorder-specific and method-specific subgroup analyses were conducted to enhance clinical interpretability.

Within the WHO EMRO context, several regional factors may further influence both the occurrence and detection of sleep-related problems in RA. Variability in access to rheumatology and sleep medicine services, limited availability of diagnostic tools such as polysomnography, and broader health-system constraints may contribute to underdiagnosis of conditions like OSA (31–33). At the same time, reliance on self-reported measures may overestimate subjective sleep impairment while under-recognizing clinically defined disorders. Cultural attitudes toward sleep, pain, and mental health, as well as differences in healthcare-seeking behavior, may also shape symptom reporting and clinical recognition. Consequently, observed prevalence estimates likely reflect a combination of true disease burden and differences in detection capacity across settings.

The findings of this review have several important clinical implications. Given the high overall burden of sleep-related problems, routine screening should be integrated into RA care using validated tools such as the PSQI, Insomnia Severity Index (ISI), and Epworth Sleepiness Scale (ESS). Patients with symptoms suggestive of OSA should be prioritized for objective evaluation where diagnostic resources are available. Management should adopt a multidisciplinary approach that addresses pain control, psychological comorbidities, and sleep-specific interventions. Evidence-based non-pharmacological strategies, particularly cognitive behavioral therapy for insomnia (CBT-I), alongside sleep hygiene education and lifestyle modification, are likely to be especially relevant in this population.

Several limitations should be acknowledged. Only a subset of WHO EMRO countries contributed data—primarily Saudi Arabia and Egypt, with additional studies from Iran and Tunisia—limiting representativeness across the entire region. The small number of included studies, variability in study design and outcome definitions, and reliance on predominantly cross-sectional data restrict causal inference and contribute to statistical heterogeneity. Some subgroup and risk-factor analyses were based on few studies, reducing statistical power and precision. In addition, study screening and data extraction were conducted primarily by a single reviewer, which may increase the risk of missed studies or extraction errors despite consultation with senior collaborators. The exclusion of non-English and non-Arabic publications and grey literature may also have introduced publication bias. Consistent with these limitations, the certainty of evidence was rated as low to very low for most outcomes using the GRADE framework.

Finally, the conceptual diversity of sleep outcomes assessed across studies should be emphasized. The included literature examined multiple, distinct sleep-related constructs—such as insomnia symptoms, OSA, and subjective sleep quality—each with different mechanisms and diagnostic criteria. Although a pooled estimate was generated to summarize overall burden, disorder-specific findings provide more precise clinical insight and should guide interpretation.

Future research should prioritize large, multicenter prospective studies across a broader range of WHO EMRO countries, using standardized and validated diagnostic tools. Improved reporting of key confounders, including disease activity, body mass index, medication use, and socioeconomic factors, is essential. Interventional studies—particularly those evaluating culturally adapted behavioral and psychosocial interventions—and longitudinal investigations examining the interplay between inflammation, disease activity, and sleep architecture are also needed to inform targeted, evidence-based management strategies.

## 5 Conclusion

Sleep-related problems—assessed using heterogeneous subjective and objective measures—are common among patients with rheumatoid arthritis in WHO Eastern Mediterranean Region (EMRO) countries with available data, reflecting a substantial overall sleep-related burden rather than a single, unified clinical diagnosis. Depression, pain, fatigue, female sex, and older age were consistently associated with worse sleep-related outcomes, although the certainty of evidence remains limited.

Given the methodological heterogeneity of included studies and the fact that only a subset of WHO EMRO countries contributed data, these findings should be interpreted with caution. Nevertheless, they underscore the importance of routine screening for sleep-related problems, multidisciplinary management addressing both physical and psychological factors, and the need for regionally tailored research and service development using standardized diagnostic approaches.
