# Supplementary material for: Sleep-related problems among patients with rheumatoid arthritis in the World Health Organization Eastern Mediterranean region: a systematic review and meta-analysis
Source: Front Psychiatry. 2026 May 29;17:1786989. doi: 10.3389/fpsyt.2026.1786989 (PMC13261355; doi:10.3389/fpsyt.2026.1786989)
Supplement: Supplementary file 2 [file SupplementaryFile2.docx]

**Supplementary file 2:** Full Database‑Specific Search Strategies (WHO EMRO)

**Geographical scope:** World Health Organization Eastern Mediterranean Region (WHO EMRO).

**Countries included:** Afghanistan, Bahrain, Djibouti, Egypt, Iran, Iraq, Jordan, Kuwait, Lebanon, Libya, Morocco, Oman, Pakistan, Palestine, Qatar, Saudi Arabia, Somalia, Sudan, Syria, Tunisia, United Arab Emirates, Yemen.

**Search period:** From inception to 27 July 2025

**Language limits:** English and Arabic

| **Database** | **Search Terms** | **Results** |
| --- | --- | --- |
| PubMed | ("Rheumatoid Arthritis"[Mesh] OR "rheumatoid arthritis" OR RA)  AND  ("Sleep Disorders"[Mesh] OR "sleep disorder*" OR insomnia OR "sleep quality"  OR "obstructive sleep apnea" OR "sleep apnoea"  OR "restless legs syndrome" OR "daytime sleepiness")  AND  (Afghanistan OR Bahrain OR Djibouti OR Egypt OR Iran OR Iraq OR Jordan  OR Kuwait OR Lebanon OR Libya OR Morocco OR Oman OR Pakistan  OR Palestine OR Qatar OR "Saudi Arabia" OR Somalia OR Sudan  OR Syria OR Tunisia OR "United Arab Emirates" OR Yemen) | 325 |
| Scopus | TITLE-ABS-KEY("rheumatoid arthritis" OR RA)  AND  TITLE-ABS-KEY("sleep disorder*" OR insomnia OR "sleep quality"  OR "obstructive sleep apnea" OR "sleep apnoea"  OR "restless legs syndrome" OR "daytime sleepiness")  AND  TITLE-ABS-KEY(Afghanistan OR Bahrain OR Djibouti OR Egypt OR Iran OR Iraq  OR Jordan OR Kuwait OR Lebanon OR Libya OR Morocco OR Oman  OR Pakistan OR Palestine OR Qatar OR "Saudi Arabia"  OR Somalia OR Sudan OR Syria OR Tunisia  OR "United Arab Emirates" OR Yemen) | 410 |
| Web of Science | TS=("rheumatoid arthritis" OR RA)  AND  TS=("sleep disorder*" OR insomnia OR "sleep quality"  OR "obstructive sleep apnea" OR "sleep apnoea"  OR "restless legs syndrome" OR "daytime sleepiness")  AND  TS=(Afghanistan OR Bahrain OR Djibouti OR Egypt OR Iran OR Iraq  OR Jordan OR Kuwait OR Lebanon OR Libya OR Morocco OR Oman  OR Pakistan OR Palestine OR Qatar OR "Saudi Arabia"  OR Somalia OR Sudan OR Syria OR Tunisia  OR "United Arab Emirates" OR Yemen) | 295 |
| Embase | ('rheumatoid arthritis'/exp OR 'rheumatoid arthritis' OR RA)  AND  ('sleep disorder'/exp OR insomnia OR 'sleep quality'  OR 'obstructive sleep apnea' OR 'sleep apnoea'  OR 'restless legs syndrome' OR 'daytime sleepiness')  AND  (Afghanistan OR Bahrain OR Djibouti OR Egypt OR Iran OR Iraq  OR Jordan OR Kuwait OR Lebanon OR Libya OR Morocco OR Oman  OR Pakistan OR Palestine OR Qatar OR 'Saudi Arabia'  OR Somalia OR Sudan OR Syria OR Tunisia  OR 'United Arab Emirates' OR Yemen) | 340 |
| CINAHL | (MH "Rheumatoid Arthritis")  AND  (MH "Sleep Disorders+" OR TX insomnia OR TX "sleep quality"  OR TX "obstructive sleep apnea" OR TX "sleep apnoea"  OR TX "restless legs syndrome" OR TX "daytime sleepiness")  AND  TX (Afghanistan OR Bahrain OR Djibouti OR Egypt OR Iran OR Iraq  OR Jordan OR Kuwait OR Lebanon OR Libya OR Morocco OR Oman  OR Pakistan OR Palestine OR Qatar OR "Saudi Arabia"  OR Somalia OR Sudan OR Syria OR Tunisia  OR "United Arab Emirates" OR Yemen) | 120 |
| Google Scholar | "rheumatoid arthritis"  AND  ("sleep disorder" OR insomnia OR "sleep quality"  OR "obstructive sleep apnea" OR "restless legs syndrome")  AND  (Afghanistan OR Bahrain OR Djibouti OR Egypt OR Iran OR Iraq  OR Jordan OR Kuwait OR Lebanon OR Libya OR Morocco OR Oman  OR Pakistan OR Palestine OR Qatar OR "Saudi Arabia"  OR Somalia OR Sudan OR Syria OR Tunisia  OR "United Arab Emirates" OR Yemen) | 37 |
